# NRSF and Its Epigenetic Effectors: New Treatments for Neurological Disease

**DOI:** 10.3390/brainsci8120226

**Published:** 2018-12-19

**Authors:** Ryan Thompson, Christina Chan

**Affiliations:** 1 Cell and Molecular Biology Program, Michigan State University, 567 Wilson Road, Rm 2240E, East Lansing, MI 48824, USA; thomp577@msu.edu; 2 Department of Chemical Engineering and Materials Science, Michigan State University, 428 S. Shaw Lane, Rm 2527, East Lansing, MI 48824, USA; 3 Department of Biochemistry and Molecular Biology, Michigan State University, 603 Wilson Road, East Lansing, MI 48824, USA

**Keywords:** neuron restrictive silencer factor, epigenetics, neurological disease

## Abstract

The Neuron Restrictive Silencer Factor (NRSF) is the well-known master transcriptional repressor of the neuronal phenotype. Research to date has shown that it is an important player in the growth and development of the nervous system. Its role in the maturation of neural precursor cells to adult neurons has been well characterized in stem cell models. While much has been characterized from a developmental perspective, research is revealing that NRSF plays a role in various neurological diseases, ranging from neurodegenerative, neuropsychiatric, to cancer. Dysregulation of NRSF activity disrupts downstream gene expression that is responsible for neuronal cell homeostasis in several models that contribute to pathologic states. Interestingly, it is now becoming apparent that the dysregulation of NRSF contributes to neurological disease through epigenetic mechanisms. Although NRSF itself is a transcription factor, its major effectors are chromatin modifiers. At the level of epigenetics, changes in NRSF activity have been well characterized in models of neuropathic pain and epilepsy. Better understanding of the epigenetic basis of brain diseases has led to design and use of small molecules that can prevent NRSF from repressing gene expression by neutralizing its interactions with its chromatin remodelers. This review will address the basic function of NRSF and its cofactors, investigate their mechanisms, then explore how their dysfunction can cause disease states. This review will also address research on NRSF as a therapeutic target and delve into new therapeutic strategies that focus on disrupting NRSF’s ability to recruit chromatin remodelers.

## 1. Introduction

The first indication of the existence of a neural repressor came from study of the sodium voltage-gated channel alpha subunit 2 gene (SCN2A) and a neuron-specific marker, superior cervical ganglion-10 (SCG10) [1,2]. The characterization of the promoter regions showed that a 21-bp neural restrictive silencer element (NRSE) was responsible for gene repression and it was bound by nuclear extracts from non-neural tissue, but not neural tissue. This led to the hypothesis that NRSE binding proteins existed and they were important for the differential expression of neural genes between neurons and non-neural cells. This *cis*-acting element would also be characterized as the RE-1 silencer and it would be characterized in a host of genes specific for neurons. The *trans*-acting transcription factor would be isolated and eventually named RE-1 silencing transcription factor (REST), or alternatively Neuron Restrictive Silencer Factor (NRSF). Further study of NRSF/REST showed that this transcription factor played a role high in the hierarchy of neuronal gene expression during development, and as such served as a master transcriptional regulator [3,4]. Since then, hundreds of genes have been identified that are regulated by NRSF/REST, and in silico studies suggest that this number could be in the thousands [5]. 

In situ hybridization has revealed that NRSF mRNA is expressed in all non-neural tissue in adult organisms [6,7]. Interestingly, NRSF seems to have a higher function in gene regulatory networks that maintain pluripotency in embryonic stem cells [8,9]. A high expression of NRSF is also present in neural stem cells to preserve stemness and prevent differentiation. Downregulation of NRSF in neural stem cells is enough to drive differentiation [10]. During the early development of the nervous system, the downregulation of NRSF de-represses gene neural expression long enough to allow for neurons to differentiate [11]. However, this downregulation during development is transient, and surprisingly, a basal level of expression in adult neural tissues is maintained throughout the life of an organism [6]. Despite what appears to be constitutive expression of NRSF, research has shown that its overall protein level does not always correlate with its *activity* level. Several factors determine if NRSF can repress expression of its target genes, including proper nuclear localization, recruitment of corepressors, and the presence or absence of dominant interfering spliced isoforms. 

While much research has focused on NRSF and the development of the nervous system, NRSF has been increasingly linked to numerous diseases involving the brain. Given the central role that it plays in neural gene regulation, this is not surprising. NRSF overexpression has been linked to brain cancers where it appears to maintain stemness of the stem cell populations within tumors [12,13,14,15]. Additionally, NRSF appears to play an increasingly important role in neurodegenerative disease (which has been well reviewed in [16]). More recently, research has implicated NRSF as an effector in the possible epigenetic basis of neurological disease [17]. Upregulation of NRSF in response to brain insults, such as ischaemia [18], is believed to be neuroprotective [19] in the short term, but may leave long term epigenetic changes that underlie neuropathic pain, epilepsy, and contribute to neurodegeneration. As these molecular mechanisms begin to be resolved, it is becoming apparent that the use of epigenetic inhibitors to target NRSF and its effector chromatin modifiers opens up the possibility for new therapeutics. 

## 2. Structure and Function of NRSF

The protein structure of NRSF is characterized and it has well defined functional domains. NRSF is a large, Kruppel-like transcription factor that contains nine zinc finger domains that control its DNA-binding specificity [3]. Being a transcription factor, the localization of NRSF/REST is important for its function. Characterization of the NRSF/REST zinc finger domains (ZFDs) revealed that in addition to DNA-binding, there is also a nuclear localization signal [20]. The generation of several deletion and truncated mutants of NRSF revealed that a nuclear localization signal (NLS) is present somewhere within the fifth N-terminal zinc finger domain. While ZFDs 6–8 appear to be most important for DNA binding, ZFD5 contains an NLS. Shimojo showed by deleting ZFD5 in NRSF/REST that REST4, which contains the first five ZFDs, is the only variant that is able to localize to the nucleus [21]. It had been suggested that amino acids 512–522 were an NLS, however, the deletion of this region formed a protein that could still localize to the nucleus. In addition to a NLS, control of nuclear import of NRSF is also dependent on the function of REST/NRSF-Interacting LIM Domain Protein, RILP [22,23]. 

NRSF binds to a conserved 21-bp sequence, termed a Neuron Restrictive Silencer Element (NRSE). After binding to DNA, NRSF represses gene expression by recruitment of repressive chromatin modifiers. The N-terminal domain of NRSF recruits the corepressor mSin3 through its paired amphipathic helix (PAH1) domain [24]. mSin3 in turn recruits histone deacetylases (HDACs) to nucleosomes to promote a chromatin repressive environment through the deacetylation of histones [25]. Separately, the C-terminal domain recruits the major corepressor, REST corepressor 1 (CoREST) [26]. CoREST itself recruits chromatin modifying enzymes, including HDACs as well as histone methyltransferases. Additionally, CoREST contains two SANT domains that allow it to interact with histones [27]. Interestingly, this can allow for the recruitment of CoREST to areas of the genome without NRSF or an NRSE and contribute to long term gene silencing, even in the absence of NRSF. Lastly, the expression of NRSF can be downregulated post-translationally through ubiquitination by B-Trcp [28]. Interaction with B-Trcp is mediated by two conserved DpSG sequences. Within this degron sequence are several critical serine residues (1024, 1027, and 1030), which, when phosphorylated, increase binding of B-Trcp to NRSF [28]. 

## 3. REST-Interacting LIM Domain Protein 

While post-translational modifications can serve as quick on-off switches, they often enable other interactions with binding partners that modulate NRSF/REST. The most studied of these is the REST-interacting LIM domain protein (RILP). While the NRSF/REST protein levels can remain constant throughout the life of a cell, its activity is far from being dependent on expression levels. Being a transcription factor, nuclear localization of NRSF is required for its function. As far as current research is aware, RILP it is one of the chief nuclear importers of NRSF. RILP directly interacts with ZFD5 of NRSF and is required for the proper differentiation and maintenance of the neuronal phenotype [23]. 

RILP is considered a nuclear envelope protein. At least three domains control this association. RILP contains a CIIS domain required for farnesylation [22,23]. Treatment of cells with farnesyl transferase inhibitor (FTI) prevents localization to the nuclear envelope. Additionally, RILP contains two domains that can be phosphorylated by PKA. Point mutations in critical phosphorylated residues also abolish RILP localization to the nucleus. Finally, RILP contains three separate NLS signals. The deletion of any single NLS abolishes localization to the nucleus, suggesting that they adapt a cooperative conformation [23]. It should be noted that one of the NLS motifs also overlaps with the phosphorylated residue of one of the PKA recognition domains. 

## 4. REST4

NRSF/REST is subjected to several splice isoforms [29]. These are driven by multiple promoters within the gene that begin expression at different exons [30]. Although the mechanism is not entirely clear, it was shown that nsr100, an important neuronal activator, promotes alternative splicing of NRSF [31]. Additionally, characterization of the cholinergic gene locus in PC12 cells showed that PKA activity may also promote REST4 splicing [32], although it is still unclear if this is a pre- or post transcriptional event. Of these isoforms, REST4 is the most studied due to its ability to antagonize NRSF function [33]. REST4 contains the first 5 N-terminal ZFDs of full NRSF. These domains, especially ZFD5, contain enough of the NLS so that REST4 can efficiently localize to the nucleus [20]. REST4 retains some function of the original NRSF protein. Structurally, REST4 is a C-terminally truncated form of the NRSF full gene. Since the C-terminal end of NRSF is known to recruit CoREST, an important co-repressor for NRSF, it is easy to imagine how REST4 could competitively inhibit full NRSF and reduce its repressive function. Indeed, part of its repressive function may be due to its ability to heterodimerize with NRSF, resulting in a complex with reduced ability to recruit CoREST [34]. However, further work by Shimojo indicates that the sixth to eighth ZFD are critical for DNA-binding. Deletion of ZFD7 plus either ZFD6 or 8 abolishes DNA-binding, implying that REST4 alone does not actually bind to DNA [21]. Since the NRSE is a 21-bp sequence and each ZFD should contribute 3bp worth of specificity, it can be logically concluded that the loss of any ZFD could decrease affinity for the NRSE. Nonetheless, despite lacking the C-terminal end, REST4 does retain a trace of its repressive function [35]. This is presumably due to the retention of the N-terminal region, which is still able to recruit mSin3. 

Alternative splicing of NRSF/REST is not comprehensively characterized. While several spliced forms have been observed, only REST4 seems to play a critical role in neural development. Inhibition of REST4 splicing leads to impairments of neurite growth and may contribute to Autism Sprectrum disorders [31]. Additionally, there are several pathologies that REST4 can be implicated in, including neuropathic pain [36], glioma [37], Parkinson’s Disease [38], and epilepsy [39]. Given the complex function of REST4 and its implication in various stages of neural development, it should not be surprising that the splicing event is also under complex regulation. 

## 5. CoREST

CoREST is a well-known co-repressor that associates with NRSF. CoREST interacts with NRSF through a single ZFD [26] and mutating this will abrogate gene repression. CoREST expands the number of gene targets that NRSF/REST regulates by several fold, in part because it is able to regulate many genes without an NRSE and regulates more genes that are not neuron-specific [40]. Additionally, CoREST contains a SANT2 [41] domain that can directly interact with histones. This allows for DNA-binding and transcriptional repression, even in the absence of a canonical NRSE/RE-1 silencer element within the gene promoter. However, most of the known CoREST regulation comprises of gene networks that are involved in neural stem cell pluripotency and its de-repression occurs during differentiation. Additionally, differential REST/CoREST complexes are involved in the differentiation of different neuronal subtypes and also control the switch between glial and oligodendrocyte subtypes [42,43]. 

CoREST is recruited to the C-terminal of NRSF/REST and it further recruits chromatin modifying enzymes, mainly HDACs and DNA methyltransferases [41], which repress gene expression. Differential expression of CoREST and NRSF/REST in the developing brain allows for another level of differential repression of neural genes [44]. More importantly, it appears that CoREST can form alternative NRSF/REST complexes that have different gene specificity as compared to NRSF/REST [45]. 

## 6. NRSF Recruits Chromatin Remodelers

REST4, RILP, and CoREST play important roles in the regulation of NRSF’s activity. However, the repressive function of NRSF is mediated by chromatin modifiers that leave repressive covalent modifications on histones and DNA. These modifications promote the formation of heterochromatin that obscures important *cis*-regulatory elements that are involved in gene transcription. 

**Figure 1:**
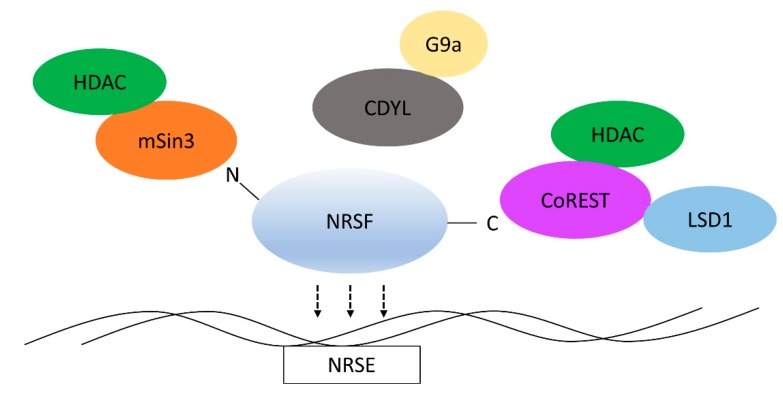
Neuron Restrictive Silencer Factor (NRSF) recruits multiple chromatin modifiers. The major function of NRSF is to recruit chromatin modifiers to neural genes by recognizing NRSEs throughout the genome. At the N-terminal end, NRSF recruits mSin3 enabling recruitment of histone deacetylase activity. Further histone deacetylase activity is conferred through recruitment of the major corepressor, CoREST, at the C-terminal end. Additionally, REST corepressor 1 (CoREST) recruits the histone demethylase LSD1. NRSF can also recruit the histone methylation activity of G9a. This is indirect through recruitment of chromodomain containing protein, chromodomain Y-like (CDYL).

To this end, NRSF relies on recruitment of HDACs, histone methyltransferases, and DNA methylases (Figure 1). NRSF recruits mSin3a to its N-terminal region [25]. From there, mSin3a itself recruits HDACs that are essential for gene repression [46]. Additionally, NRSF recruits the histone methyltransferase, G9a [47]. This interaction is indirect and it partly depends on NRSF’s recruitment of the chromodomain containing protein, chromodomain Y-like (CDYL) [48]. G9a seems to preferentially demethylate H3K9 [48], and this activity is non-overlapping with HDAC repression from either mSin3a or CoREST. Lastly, CoREST itself acts as a HDAC recruiter [41]. CoREST, through interactions with methyl CpG binding protein 2 (MeCP2) [49], may also mediate long-term gene repression by binding to methylated DNA. 

## 7. NRSF-Related Diseases

### 7.1. Epilepsy

Epilepsy is a collection of seizure disorders characterized by uncontrolled electrical activity in the brain leading to confusion, loss of consciousness, and uncontrolled movements [50]. At least one connection from NRSF has a heritable genetic component. Mutation in RILP causes the mislocalization of NRSF in Progressive Myoclonus Epilepsy-Ataxia Syndrome [51]. In a study of myoclonus epilepsy-ataxia syndrome, it was found that several families had a mutation in the NRSF translocator, RILP (called PRICKLE1 in the study). When this mutation was cloned in vitro, it was found that it caused mislocalization of NRSF and kept it nuclear instead of cytosolic. Mutations that interfere with the RILP-NRSF association underlie several brain pathologies. A mutation in RILP (R104Q) across three families with progressive myoclonus epilepsy (PME) was determined to be a founding mutation in all cases. This mutation prevents the association of RILP with NRSF allowing NRSF to accumulate in the nucleus. The dysregulation of NRSF seems to be implicated in epilepsy, however, specific mechanisms are still lacking.

REST4 may have another indirect connection to epilepsy. During seizure, NRSF and REST4 are both upregulated. However, the expression of the proconvulsant gene, TAC3 (neurokinin B (NKB)) is upregulated [52]. It appears that the increase in REST4 expression may competitively inhibit the repression of NKB by NRSF. This effect is decreased if the anticonvulsant, carbamezapine, is administered. Taken together, this suggests that the disruption of the mechanisms that control NRSF can also underlie pathologies that are associated with NRSF. 

The expression of several ion channels and receptors have been identified to contribute to the aetiology of epilepsy. Not surprisingly, these channels are critical for electrical signaling between neural cells. In particular, the dysregulation of expression of Na+ and K+ channels is highly associated with epilepsy. The molecular mechanisms that underlie these changes in gene expression are still under investigation, but it is clear from some studies that many of the genes can be directly regulated by NRSF [53]. Dysregulation of the ion channel genes SCN2A, potassium voltage-gated channel subfamily Q member 2 (KCNQ2), and KCNQ3 contribute to the progression of epilepsy in infants, but more interestingly, these are known to be repressed by NRSF [3,54]. Adding to this list, mutation in SCN1A and SCN1B contribute to an inherited form of febrile seizures in early childhood and are also direct targets for NRSF repression [55]. Another important factor for NRSF induced epilepsy is the regulation of potassium channels through epigenetic repression. Specifically, the DNA dimethylase G9a, has been shown to leave the repressive histone mark H3K9me2 on several genes for potassium channels [56].

Aside from traumatic brain injury, seizure itself can promote epilepsy in adults. In both cases, injury causes the downregulation of expression of important genes that are implicated in epilepsy, of note, the hyperpolarization-activated cyclic nucleotide-gated ion channel gene, HCN [57,58]. This relationship between NRSF and epilepsy has also been established in in vivo models of epilepsy. Therapeutic targeting of NRSF to restore HCN expression can slow down the progression of epilepsy after injury [57] in mouse models. 

Interestingly, NRSF may play a role in diet-based resistance of epilepsy. In a dietary model of epilepsy treatment, the deprivation of glucose leads to reduced NADH activation of the chromatin modifier CtBP [59]. The effect reduced NRSF repression specifically of the BDNF gene. 

### 7.2. Neuropathic Pain

Injury to nerves in the form of ischemia, crushing/mechanical, and inflammation often leave lasting symptoms. Nerve damage can result in neuropathic pain, a condition where pain thresholds to common stimuli are lowered and analgesic effects are attenuated [60]. Neuropathic pain decreases the quality of life of the injured and can lead to disability. At the cell biology level, neuropathic pain is linked to aberrant expression of ion channels and G-protein coupled receptors. Interestingly, many of these channels that are dysregulated in neuropathic pain are the same channels that are dysregulated in epilepsy [61,62,63,64], in particular, sodium and potassium channels. However, the expression pattern of these genes is very different as many of the changes occur in the peripheral nervous system. Additionally, some changes in gene expression are directly responsible for mediating the analgesic response. Most notably are changes in the mu-opioid receptor (MOR) [65,66]. Given the roles of these channels in nerve signaling and analgesia, respectively, these are logical targets for treatment. Common treatments for neuropathic pain are often tricyclic antidepressants, serotonin and norepinephrine reuptake inhibitors, gapabentin, and less commonly, opioids. These can lose their effectiveness over time as the user builds a tolerance and can carry a high risk for addiction. 

Downregulation of several types of ion channels that are commonly seen during nerve damage is confirmed in in vitro models. Additionally, other genes involved in maintaining analgesia, mainly the µ-opioid receptor are also affected and can contribute to pain. Expression of NRSF is upregulated during the same injuries that cause neuropathic pain [67]. Given its master role in regulating neural expression, it is not surprising that other laboratories have shown that the repressive effect of NRSF may be responsible for the downregulation of ion channels and analgesic promoting genes that underlie neuropathic pain. Work by Uchida et al. has shown that the sodium and potassium channels, sodium channel protein type 7 subunit alpha (SCN7A, aka Nav)2.1, and potassium voltage-gated channel subfamily D member 3 (Kv4.)3 [68] are downregulated in dorsal root ganglia after injury, possibly lowering the firing threshold for pain, while MOR expression can be directly repressed by NRSF [69,70], removing important analgesic relief. Damage to the C-fiber nerves is highly implicated in neuropathic pain and is attributed to NRSF repression of Nav1.8 and MOR genes. G9a also contributes to long-term pain through downregulation of potassium channels by methylation of histones [56]. Logically, NRSF itself can be recognized as a therapeutic target and in some pain models, since the direct inhibition of its activity has been shown to reduce symptoms [71]. Further study into the epigenetic mechanisms that are perturbed in neuropathic pain can provide more finely resolved targets. In particular, targeting the co-repressors and epigenetic effectors that NRSF recruits during injury could better treat neuropathic pain and limit off target effects. 

## 8. Epigenetic Inhibitors 

Epigenetic treatments for disease are increasingly being investigated for a range of diseases. Valproic acid, an organic acid with pan-HDAC inhibiting function, is currently in use to treat seizure [72] and bipolar disorder [73]. However, due to its broad range, pan-HDAC inhibitors are associated with many side effects. Interestingly, HDAC inhibitors are being put forth as a new potential treatment for neuropathic pain [74]. These can effectively ameliorate symptoms of pain and show distinct epigenetic changes in gene regulation. Given the known regulation of ion channels and other pain receptors by HDAC recruitment of NRSF, one could hypothesize that the global use of HDAC inhibitors could be narrowed down to a subset of NRSF regulated genes. In order for this to be tested, further research should establish a more direct link between the HDAC recruitment ability of NRSF and neuropathic pain itself. The possibility that epigenetic changes specific to neuropathic pain and NRSF could help to narrow down therapeutic options from that of global HDAC inhibition to more targeted NRSF regulated genes is exciting and it could result in therapies with less off-target effects. 

**Figure 2:**
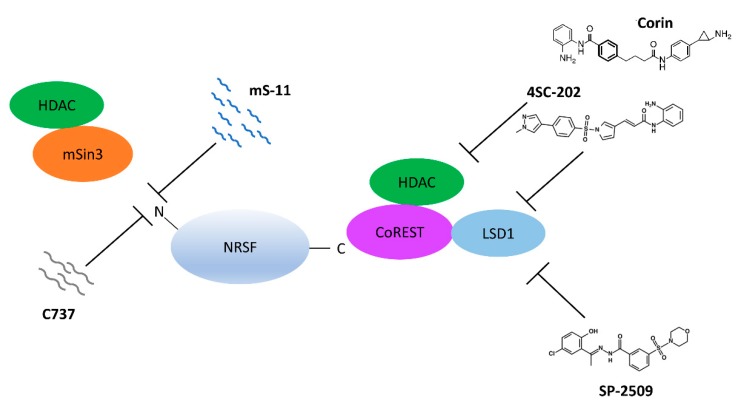
Inhibiting the Chromatin Modifying Effectors of NRSF. Inhibition of mSin3 recruitment by NRSF at the N-terminal end has been shown using molecules that mimic the helix structure of NRSF that recruits mSin3. At the C-terminal end of NRSF, small molecules against the major corepressor of NRSF, CoREST, can inhibit both deacetylase and demethylase activity.

Research on the mechanisms that show NRSF and its relationship to disease may offer the possibility to create better drugs that more precisely target the epigenetics in neural cells (Figure 2). Mimetics against NRSF are being constructed and tested in pain models. In particular, two mimetics that compete with the N-terminal end of NRSF have been synthesized under the PRISM model in Japan and tested in animals. Both mS-11 and C737 have been made to outcompete mSin3 and attenuate the repression of gene expression. In a mouse pain model for sciatic nerve injury, the administration of mS-11 was able to restore C-fiber pain stimulation threshold to basal levels [75]. In a cold stress model, shrews were subjected to depression via exposure to cold temperature. Administration of C737 therapeutically protected against cold stress-induced weight loss and performed even better when compared against the antidepressant agomelatine [76]. These mimetics highlight a new strategy where specific epigenetic effectors that associate with transcription factor activity can be inhibited. Although both seem promising, much more work with mimetics such as these needs to be performed to show that mimetics can restore homeostasis of gene expression underlying neuropathic pain. In addition to mimetics, the small quinolone-like compound 91 (C91) was tested and revealed to inhibit the NRSF-mSin3b interaction [77]. This was tested in a Huntingtin Disease model, where it was shown to restore expression of BDNF among other neural genes [77]. 

While mimetics have been synthesized that target the N-terminal domain of NRSF, small molecules have also been tested against the C-terminal domain’s interaction with CoREST. The small molecules 4SC-202 and SP2509 were able to inhibit the deacetylase and demethylase activity of the NRSF/REST-CoREST complex in medullablastoma cells, and they negatively affected cell viability [78]. The synthetic HDAC inhibitor, corin, was also shown to have dual inhibitory activity against the deacetylase and demethylase activity of the CoREST complex [79]. Interestingly, although each molecule inhibits HDAC1, they have differential affinity towards different HDAC1-containing complexes. 

## 9. Conclusions

Decades of study of the master transcriptional regulator, NRSF, has highlighted its importance in neural development. NRSF controls one of the most complex expression programs in development. The number of binding partners and effectors that have been characterized as essential for its function speaks to its complexity. Its implication in a range of neurological diseases underscores how critical its tight regulation is for cellular homeostasis. 

However, the study of this transcriptional repressor is still incomplete and further elucidation of its functional mechanisms could provide new therapeutic windows into neural dysfunction and disease. Targeting NRSF activity with siRNA has been shown to induce differentiation and reduce tumor progression in glioblastoma models, however, this approach may be too broad for use under physiological conditions. Therefore, the study of the epigenetic regulators and co-repressors that NRSF utilizes could provide a higher level of resolution for more targeted treatments.
